# Clinical utility and future direction of speaking valve: A review

**DOI:** 10.3389/fsurg.2022.913147

**Published:** 2022-09-08

**Authors:** Suna Lian, Liying Teng, Zhi Mao, Hongying Jiang

**Affiliations:** ^1^ High-Dependency Care Unit, Beijing Rehabilitation Hospital, Beijing, China; ^2^ Department of Critical Care Medicine, Chinese People’s Liberation Army General Hospital, Beijing, China

**Keywords:** valve, tracheostomy, intensive care unit, mobility, review

## Abstract

This paper summarizes and analyzes the clinical research progress of the speaking valve in recent years, including the structure and function of the speaking valve, the impact of the speaking valve on the patient’s vocalization or speech, the impact on ventilator weaning and tracheal intubation and extubation, and the effect on aspiration and swallowing function, the impact on patient mobility and quality of life. Related issues in clinical use are also described.

## Introduction

Critically ill patients often require tracheostomy, endotracheal intubation, and mechanical ventilation with a ventilator. However, tracheotomy destroys the normal structure of the airway, causing changes in the path of gas in and out, which brings a series of adverse effects to patients. In particular, the lower respiratory tract is directly connected to the outside world, resulting in the disappearance of the subglottic pressure and the loss of the patient’s vocal function. Moreover, the change of airway resistance leads to the retrieval or even disappearance of the normal protective physiological functions of the human body, manifested as dysphagia, weakened cough reflex, increased secretions in the oral and nasal cavity, loss of smell, etc. To this end, scholars have researched and developed the speaking valve. After continuous development, this invention has been widely used in clinical practice. After decades of use, the speaking valve can not only improve the patient’s vocalization and swallowing function, but also achieve certain results in various aspects such as reducing respiratory secretions and reducing aspiration. This article summarizes and analyzes the research progress of the speaking valve in clinical application in recent years.

## Methods

Relevant literatures were searched in database including PubMed, Medline and Embase. The search words include: speaking valve(s), speech valve(s), Passy-Muir valve. The publication time of literatures is from inception to Nov 14, 2022. This review was conducted according to the Preferred Reporting Items for Systematic Reviews and Meta-Analyses (PRISMA) guideline (1).

## Speaking valve: structure and function

**Figure 1:**
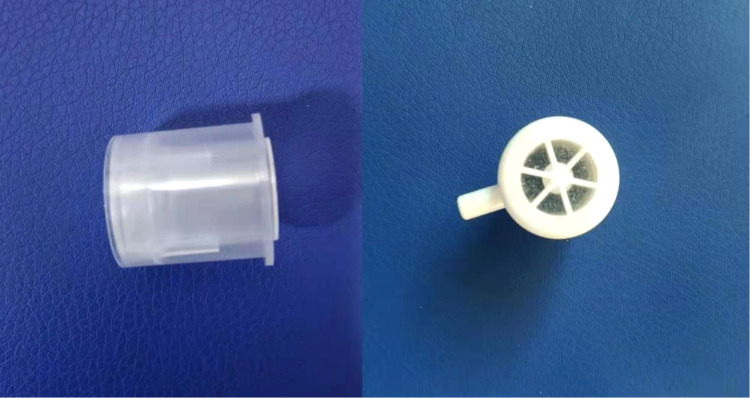
A speaking valve often used in our clinical practice.

**Figure 2:**
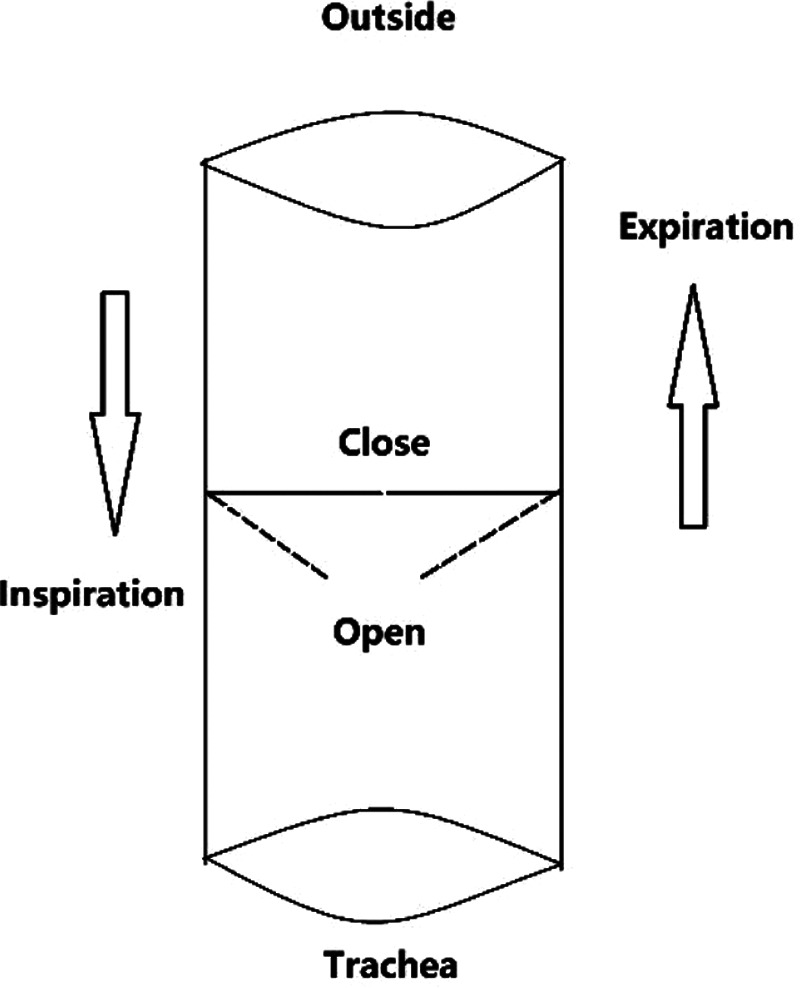
Major working principle of speaking valve. When inhaling, the valve opens, air or ventilator gas enters the trachea, and when exhaling, the valve closes, and the airflow forms a certain pressure on the catheter and the upper airway, forcing the gas to be discharged from the upper airway.

In the intensive care unit, the speaking valve used in patients underwent tracheotomy, also known as the voice valve, was first introduced by Toremalm in 1967 (2). At present, the most commonly used speaking valve in the world is the Passy-Muir swallowing and speaking valve (PMV) improved by Passy et al. (3–5). The speaking valve is generally made of silicone and is essentially a one-way closed ventilation valve that is installed at the entrance of the tracheal cannula (Figures 1, 2). When the patient inhales, the valve opens, and the ventilator or external airflow enters the airway through the opening of the valve to complete the inspiratory function. When the patient exhales, the valve closes, and the air is exhaled from the tracheal tube and the tracheal space through the upper airway (6, 7). The speaking valve was originally used to improve swallowing and speaking in tracheotomy patients (8). According to the working principle of the speaking valve the inhalation process of the patient does not change after wearing the speaking valve, but when exhaling, it no longer passes through the tracheal cannula, but exhales through the upper airway through the gap between the tracheal cannula and the trachea. Air travels through the vocal cords, expelling it from the nose and mouth, thereby remodeling the patient’s subglottic pressure (9), thus restoring upper airway airflow, improves throat sensation, rebuilds glottis closing reflex and cough reflex, restores intrapharyngeal pressure, enables patients to restore vocalization, speaking and swallowing functions, and reduces the risk of leakage and aspiration (6–8). For patients who cannot tolerate total tracheostomy occlusion, or who cannot be extubated for a long time after tracheostomy, the speaking valve can be used as a transitional method for the occlusion process.

## Speaking valve on phonation or speaking

Normal human speech requires a certain amount of pressure in the pharynx, but in patients with tracheotomy the pressure difference between the inside and outside of the glottis disappears due to the rerouting of the airflow in and out, resulting impaired voice. Although it is possible to communicate with patients in other ways, it is still easy to cause communication difficulties and inaccurate information transmission. At the same time, due to the inability to vocalize, patients will develop to a certain degree of anxiety and psychological burden, which affects the treatment effect. Therefore, restoring the patient’s voice in a timely manner is conducive to more accurate communication between medical staff and patients, and restores the patient’s confidence in treatment. After wearing the speaking valve, the pressure in the pharyngeal cavity is immediately restored, and the patient can quickly resume vocalization, which brings obvious psychological encouragement to the patient, and is more conducive to breathing training.

For patients receiving mechanical ventilation of ventilator, PMV can not only be directly linked with the tracheal cannula, but also with the ventilator tube, so that the patient can speak when using the ventilator, but at this time the medical staff is required to adjust the ventilator parameters appropriately, so as to find the appropriate ventilator usage parameters.

Passy et al. observed the effect of the speech valve on the patient’s language function (7). The results showed that the speech valve could improve speech intelligibility, speech flow, reduce speech hesitancy, and prolong speech time in patients (7). Freeman-Sanderson et al. observed the effect of early use of a speaking valve on vocalization in patients undergoing endotracheal intubation and mechanical ventilation. At the time of the final analysis, 15 patients were included in the intervention group and 11 patients in the control group. The results showed that the early use of the speaking valve allowed the patients in the intervention group to recover vocalization for an average of 7 days, compared with 18 days in the control group. Patients in the intervention recovered vocalization an average of 11 days earlier than in the control group (10). In another study, Sutt included mechanically ventilated patients and analyzed the impact of the speaking valve on the time it took to resume verbal communication from the start of tracheotomy, and found that the average time required for patients using the speaking valve was 9 days, while patients without the speaking valve took an average of 18 days (11). In a prospective study with a small sample size, Manzano et al. also found that PMV can improve patients’ verbal communication skills (12).

## Speaking valve on decannulation and mechanical ventilation

In some critically ill patients, ventilator weaning and tracheal cannula extubation are at greater risk. How to shorten the time of ventilator use and increase the safety of ventilator weaning is the difficulty and end point of current research. In the study of Freeman-Sanderson et al., the time of extubation was 1 day longer in patients with early intervention using the speaking valve than in the control group, but there was no statistical difference (Hazard ration = 1.40, 95%CI: 0.65–3.03) (10). They also found that the patients who used the speaking valve intervention earlier used mechanical ventilation an average of one day less than the control group, but the difference was still not statistically significant (10). Similarly, the researchers retrospectively analyzed the effect of the speaking valve on the duration of mechanical ventilation. In a retrospective study, Sutt et al. found that the use of the speaking valve did not prolong the duration of mechanical ventilation and extubation of endotracheal intubation (11). In another prospective observational study, they found that wearing a speaking valve significantly increased tidal volume compared with baseline (13), and that the use of PMV facilitated lung recruitment during weaning from the ventilator (14).

## Speaking valve on aspiration and swallowing

In general, tracheostomy is a treatment used when the patient is unable to breathe on his own or when the airway is obstructed by a high volume of sputum. Although tracheotomy can ensure the smooth breathing of patients, it will also bring a series of physiological and functional changes (15), including reduced or even disappearance of airway resistance, failure to form subglottic pressure during swallowing, reduced muscle sensitivity, weakened vocal cord closure and coordination, weakened cough reflex, and weakened laryngeal lift (16). At the same time, after tracheotomy, the lower respiratory tract is directly connected to the outside world, and the gas entering the lower respiratory tract does not have the functions of moistening, humidifying, and screening out micro-particles in the upper respiratory tract, which causes the secretions of the respiratory tract mucosa to thicken, harden and even become thicker on the tracheal wall, and blocks small bronchi, resulting in obstruction of secretions discharge (16). Moreover, studies have found that after tracheotomy, patients have reduced lung compliance and decreased lung function. These factors together make patients vulnerable to aspiration and aspiration pulmonary infection after tracheotomy (17). In addition, because of impaired consciousness or brain function and throat function, most patients with tracheotomy have difficulty swallowing and are prone to aspiration, which further increases the risk of aspiration pneumonia (18).

A study by Dettelbach et al. observed the effect of PMV on aspiration of patients while eating, and the results showed that wearing PMV could reduce or even prevent aspiration regardless of whether patients ate liquid, semi-liquid or solid food (19). The study by Lichtman et al. found that the use of the speaking valve can significantly reduce the accumulation of tracheal secretions, but has no significant effect on 24-hour arterial oxygen saturation. The results also showed that the use of the speaking valve can improve the patient’s sense of smell (20). Manzano et al. also found in a study that the use of PMV can reduce secretions in the respiratory tract and improve cardiopulmonary function (12). Passy et al. found that patients who used the speaking valve had significantly less oral and nasal secretions and significantly less suctioning by nursing staff (7). The findings of Elpern et al. have similar findings (21). Using a more accurate video-fluoroscopy swallow test, they looked at patients’ aspirations while drinking thin liquids, and showed that wearing a PMV can significantly reduce aspiration (21). The study by Sutt et al. also compared the effect of the use of the speaking valve on swallowing function and found that although the use of the speaking valve had no significant effect on the recovery time of fluid or food intake, patients using the speaking valve needed to take thicker fluids, and 42% of patients using the speaking valve were able to consume food while the tracheal tube balloon was deflated, while those who did not all needed to eat while the balloon was inflated (11).

## Speaking valve on mobility and quality of life

In critically ill patients, early mobilization can prevent ICU-related muscle wasting and decline in physical function (22, 23). Ceron et al. conducted a cohort study of patients undergoing tracheostomy who were weaning from mechanical ventilation (24). The study ultimately included 18 patients, whose mobility status was assessed using daily measurements of the Perme Intensive Care Unit Mobility Score. The results showed that the patient’s Perme score increased rapidly from 11.3 (10.1–12.0) before wearing the speaking valve to 18.2 (16.2–20.1) after wearing the speaking valve for 1 day (*P* < 0.01). The authors further determined that the speaking valve improved the abilities of “sit to stand,” “static standing balance once standing position is established,” and “transfer from bed to chair OR chair to bed” (24). There are also studies looking at the impact of the speaking valve on the quality of life of patients with endotracheal ventilator-assisted breathing. The researchers used the visual analog self-esteem scale (VASES) and the EuroQol-5D questionnaire (EQ-5D) to assess the patients’ quality of life (10). The results showed that the between-group differences in the seven indicators of VASES suggested that the early use of the speaking valve might be beneficial to improve the quality of life of patients, but there was no statistical difference; similarly, the EQ-5D results also suggested that the early use of the speaking valve might improve the quality of life, but there was also no statistical difference (10). These results may be related to the small sample size included in the final analysis, and subsequent studies should include larger sample sizes for analysis.

## Speaking valve use in clinical practice

Although the invention of the speaking valve has a history of decades, it has also been widely used in European and American countries. In China, especially the mainland and some other countries, there are still many patients who do not use the speaking valve. Some research analysis believes that it may be related to the lack of multidisciplinary cooperation. In a meta-analysis, Speed et al. found that multidisciplinary collaboration can significantly improve the use of speaking valves (25) with a total of 3 studies were included in this analysis. In these 3 studies, the use rates of speaking valves were 35%, 33%, and 19.4% before multidisciplinary cooperation was adopted, and after multidisciplinary cooperation, the use rates of speaking valves reached 82%, 71% and 67.4%, respectively (26–28). At the same time, multidisciplinary collaboration can also shorten the time to start using the speaking valve (25, 26). An analysis by Martind et al. compared standard implantation of a speaking valve (speaking valve implantation within 48–60 h of completion of tracheostomy) with accelerated implantation (speaking valve implantation within 12–24 h of completion of tracheotomy), 10 patients were included in each of the two groups, and it was found that it was feasible to implant the speaking valve within 24 h after the completion of the tracheotomy, and no adverse events such as aspiration and hypoxemia occurred in the two groups. There was no significant difference in speech intelligibility between the two groups. Also, at the end of the study, patients in the accelerated implant group using the speaking valve significantly longer than the standard implant group, and more patients had their cannula removed at discharge (29). There are also studies looking at the safety and efficacy of prolonged use of the speaking valve (30). The study by O’Connor et al. found that in patients who used the speaking valve for a total time of more than two hours and a maximum of 17 h, there were no significant changes in cardiopulmonary function indicators, and no significant adverse events occurred (30). In their study, Freeman-Sanderson et al. also assessed the impact of early use of a speaking valve on length of stay in the ICU. The results showed that there was no statistical difference in the length of hospital stay between the intervention group and the control group (10).

## Conclusion and future direction

The speaking valve can quickly improve the patient’s vocalization and swallowing function, while reducing problems such as aspiration. It has good safety in clinical application. According to the current research results, it is recommended that in patients with indications one can consider the early use of the speaking valve. However, there are few studies related to the prognosis of patients with the speaking valve. Future studies should conduct follow-up studies on the short-term prognosis of patients during hospitalization and long-term prognosis after discharge, so as to provide more abundant evidence for the standardized use of the speaking valve.
